# The Relationship between Vitamin D Status and Rotator Cuff Muscle Strength in Professional Volleyball Athletes

**DOI:** 10.3390/nu11112768

**Published:** 2019-11-14

**Authors:** Do Kyung Kim, Geon Park, Liang-Tseng Kuo, Won Hah Park

**Affiliations:** 1 Department of Sports Medicine, Samsung Medical Center, Sungkyunkwan University School of Medicine, Seoul 03063, KoreaGeon2.park@samsung.com (G.P.); 2 Department of Orthopaedic Surgery and Sports Medicine Center, Chang Gung Memorial Hospital, Chiayi 613, Taiwan; 3 School of Medicine, College of Medicine, Chang Gung University, Taoyuan 333, Taiwan

**Keywords:** vitamin D, muscle strength, volleyball, athletes, shoulder

## Abstract

This study aimed to examine the vitamin D status of professional volleyball athletes and to determine its correlation with shoulder muscle strength. We included 52 healthy male professional volleyball players (23.2 ± 4.5 years), who were categorized by vitamin D status (<20 ng/mL: deficiency, 20–30 ng/mL: insufficiency, and >30 ng/mL: sufficiency). We examined the strength of the internal rotator (IR) and external rotator (ER) muscles of the shoulder by using an isokinetic dynamometer. Fourteen players (26.9%) had vitamin D deficiency, 24 players (46.2%) were vitamin D-insufficient, and 14 players (26.9%) were vitamin D-sufficient. There was no significant correlation between vitamin D level and shoulder muscle strength at 60°/s (IR, *r* = 0.159, *p* = 0.26; ER, *r* = 0.245, *p* = 0.08) and at 180°/s (IR, *r* = −0.093, *p* = 0.51; ER, *r* = −0.037, *p* = 0.79). Moreover, the isokinetic shoulder strengths were not significantly different across the three groups in all settings. In conclusion, vitamin D insufficiency was common in elite volleyball players. Though not being associated with isokinetic muscle weakness, vitamin D levels should be regularly monitored, and vitamin D should be supplied to young elite athletes, considering its importance for musculoskeletal health.

## 1. Introduction

Vitamin D is necessary for calcium balance and bone metabolism [1]. Vitamin D deficiency is associated with sarcopenia, impaired muscle actions, and decreased muscle strength [2,3]. In a health check-up survey in Korean colleges, more than 80% of undergraduates were found to have vitamin D insufficiency or deficiency [4]. Furthermore, a systematic review of 2000 young athletes reported that 56% had inadequate vitamin D levels [5,6].

Vitamin D is of particular interest to athletes, since the injury rate may decrease with increasing vitamin D levels [7]. Meanwhile, vitamin D is also useful in skeletal muscle repair and remodeling [8]. Further, it is also known to facilitate the recovery of the muscles after high-intensity exercises [8]. These findings imply that adequate vitamin D exposure can modify the acute adaptive response to damaging physical work [8,9,10]. A previous systematic review on the effect of vitamin D on muscle functions and strength had shown that muscle strength in individuals with vitamin D deficiency improved when vitamin D levels were elevated by using supplements [11]. Furthermore, studies in older adults consistently reported that vitamin D had positive effects on muscle strength and functions [12,13]. More recently, vitamin D has shown to have a direct impact on the skeletal muscles through the vitamin D receptor, recognizing that vitamin D may play a role in muscle function and recovery and potentially in physical and athletic performances [14]. Although the importance of vitamin D for athletes had been emphasized, the results are conflicting among studies [15,16,17,18]. Some studies reported that increasing vitamin D levels could increase quadriceps strength and enhanced vertical jump and sprint performance in athletes with vitamin D insufficiency [15,18], but other studies found that vitamin D levels were not associated with muscle strength and function [16,17]. Therefore, the effect of vitamin D levels on muscle function and sports performance remains uncertain.

Thus, we designed the current study to elucidate the relationship between vitamin D level and muscle function. This study aimed to examine the vitamin D status of professional volleyball athletes and to investigate its associations with age, height, body weight, body mass index (BMI), and isokinetic muscle strength including external rotation (ER) and internal rotation (IR) strength of the shoulder.

## 2. Materials and Methods

### 2.1. Subjects and Demographics

This retrospective study was conducted on players of a professional volleyball team. From January 2014 to December 2018, we enrolled in the study 52 players, all of whom were medically cleared for participation by an orthopedic specialist. We excluded players with any shoulder surgical history and those taking vitamin D supplements concurrently. All research procedures were reviewed and approved by the bioethical committee of Sungkyunkwan University; the study conformed to the tenets of the Declaration of Helsinki for medical research involving human subjects (IRB no: 2019-03-120).

### 2.2. Assessment

Vitamin D sufficiency is best determined by measuring the serum 25-hydroxyvitamin D [25(OH)D] level. Herein, the serum 25(OH)D level was measured using the Elecsys Vitamin D Assay (electrochemiluminescence binding assay for in vitro determination of total 25(OH)D; Roche Diagnostics, Risch-Rotkreuz, Switzerland). Participants were divided into groups according to vitamin D level: deficiency group, <20 ng/mL; insufficiency group, 20–30 ng/mL; and sufficiency group, >30 ng/mL [3,12,19].

We evaluated the IR and ER strengths and peak torques of the bilateral shoulder muscles using an isokinetic dynamometer (CSMI Medical Solutions, MA, USA). All tests were measured by the same qualified person, who was familiar with a Cybex CSMI isokinetic dynamometer, to ensure the quality and reliability of the test. Concentric shoulder ER and IR peak torques were measured at angular velocities of 60°/s and 180°/s. The participants performed three submaximal familiarization trials. Thereafter, they underwent maximal concentric IR and ER strength tests. We provided standardized and consistent oral encouragement such as “push as hard as possible” and “push as fast as possible.” After a 5 min break, the test was repeated on the other shoulder using the same protocol [20]. The peak torques (PT) generated from the isokinetic dynamometer were expressed as ft/lb.

### 2.3. Statistical Analysis

All statistical analyses were conducted using SPSS version 18.0 (SPSS Inc., Chicago, IL, USA). We evaluated the correlation between the player parameters and vitamin D level using Pearson correlation coefficients. We also compared the concentric ER/IR isokinetic strength of the dominant shoulder between three groups by using one-way ANOVA. Post-hoc Bonferroni test was used to compare the intergroup difference. The level of statistical significance was set at 0.05. 

## 3. Results

As shown in Table 1, the mean age of the participants was 23.8 ± 2.8 years (range, 19–32 years). The mean vitamin D level was 25.2 ± 8.3 ng/mL (range, 8.9–44.9 ng/mL). Fourteen players (26.9%) had vitamin D deficiency; 24 players (46.2%) had vitamin D insufficiency, and the remaining 14 players (26.9%) had vitamin D sufficiency. A total of 38 players (73.1%) had either vitamin D deficiency or insufficiency.

Table 2 shows the Pearson correlation analysis of the relationship between the athletes’ physical characteristics and vitamin D levels. There was a significantly negative correlation between age and vitamin D level; the vitamin D level decreased with increasing age (*r* = −0.315, *p* = 0.02, Table 2). The vitamin D levels were not associated with other variates, including height, body weight, and BMI. The analysis of the correlation between the vitamin D level and shoulder ER/IR strength showed no significant findings. No significant correlation was detected between the vitamin D level and shoulder ER and IR strength at the angular velocity of 60°/s (*r* = 0.159, *p* = 0.26 and *r* = 0.245, *p* = 0.08, respectively, Table 2) and at the angular velocity of 180°/s (*r* = −0.093, *p* = 0.51 and *r* = −0.037, *p* = 0.79, respectively, Table 2).

**Figure 1:**
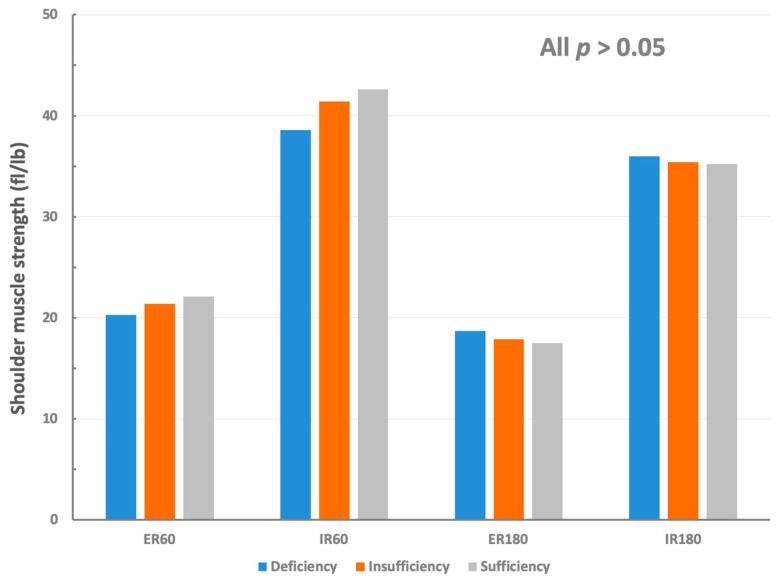
Internal and external shoulder muscle strength according to the vitamin D status. The isokinetic shoulder strengths were not significantly different across the three groups in all settings. (ER60, external rotation at 60°/s; IR60, internal rotation at 60°/s; ER180, external rotation at 180°/s; IR, internal rotation at 180°/s).

Furthermore, the participants were divided into three groups to examine physical and functional differences according to the vitamin D levels (Table 3). There were no significant differences between the three groups with respect to physical characteristics, except age. There were also no significant differences between groups regarding isokinetic shoulder muscle strength (Figure 1, Table 4). 

## 4. Discussion

In this study, we examined the vitamin D levels and isokinetic shoulder strengths of male professional volleyball players. Our findings showed that 73% of the athletes were either vitamin D-insufficient or -deficient. However, participants with vitamin D deficiency and insufficiency did not have a significantly weaker isokinetic shoulder muscle strength than those with sufficient vitamin D.

Vitamin D insufficiency or deficiency was not uncommon in elite athletes. In a study on 279 National Basketball Association (NBA) players, 79.3% of the athletes had vitamin D insufficiency or deficiency, of which 90 had vitamin D deficiency (32.3%), and 131 had vitamin D insufficiency (47.0%) [19]. In another investigation on 80 National Football League (NFL) players, 68.8% of the athletes were vitamin D-insufficient or -deficient, with 21 (26.3%) having vitamin D deficiency and 34 (42.5%) having vitamin D insufficiency [21]. Similarly, 73.1% of the professional athletes in our study had vitamin D insufficiency or deficiency, suggesting that inadequate levels of vitamin D are not rare in athletes. 

According to the 2008–2013 report of the Korea National Health and Nutrition Examination Survey (KNHANES), the vitamin D levels of 34,587 individuals in the general Korean population ranged from 16.8 to 19.4 ng/mL [4]; the mean vitamin D levels in our study population (i.e., volleyball athletes) was 25.2 ± 8.3 ng/mL. These findings indicate that athletes have a higher vitamin D level than the general population. This is similar to previous findings that the vitamin D levels are relatively higher among athletes than among the general population [7,15]. This is because athletes are more exposed to sunlight during training; further, athletes practicing indoor sports are known to have lower vitamin D levels than those practicing outdoor sports [6,15]. Sunlight, particularly ultraviolet radiation, is crucial for vitamin D synthesis. Athletes practicing indoor sports are relatively less exposed to sunlight and thus have a lower vitamin D level, and there are also seasonal effects on vitamin D owing to the varying seasonal amounts of sunlight exposure [22]. Moreover, many studies report that older individuals are more likely to have vitamin D insufficiency because they engage less in outdoor activities that expose them to sunlight and have reduced ability for vitamin D synthesis in the skin and calcium absorption in the intestine [3,11]. Our study also found that vitamin D is associated with age but not with height, body weight, or BMI.

Whether vitamin D impacts the musculoskeletal function of athletes is still controversial. Although the importance of vitamin D in the muscle function of athletes has been proposed, there are only a few studies conducted on athletes, and most discussions are focused on data from non-athletes. In an assessment of muscle strength, including grip strength, in healthy young adults after vitamin D supplementation who performed single-repetition maximum bench press and single-repetition maximum leg press, vitamin D was found to have a positive effect on muscle strength [23]. Moreover, Close et al. reported that increasing vitamin D intake for eight weeks in athletes and non-athletes could decrease 10 m sprint times and enhance exercise abilities, such as vertical jump performance [15,21]. This suggests that vitamin D facilitates the transportation of calcium in the sarcoplasmic reticulum via enhancing the efficiency of calcium binding during skeletal muscle contraction [7]. As a result, the supply of vitamin D increased the size and amount of type II (fast-twitch) muscle fibers, thereby impacting muscle strength [7,8,24]. However, in a study on 314 professional soccer athletes who performed isokinetic exercises, there was no association between lower-limb muscle strength and vitamin D levels [16]; another study also reported that the associations of muscle strength and exercise abilities with vitamin D levels in athletes could not be adequately explained [17,25].

Vitamin D insufficiency affects exercise performance and injury prevention; thus, it is an essential topic of interest for athletes. Moreover, it is also known to impact athletic performance; however, most previous studies have assessed jumping and running performances [26]. Thus, our study is the first study to analyze the association between vitamin D levels and shoulder rotator cuff strength, which is involved in one of the most forceful movements in volleyball, i.e., spiking. In our study, vitamin D levels did not affect the rotator cuff strength. A recent Korean study [27] on adolescent athletes also showed that vitamin D status did not show any significant effects on performance factors or blood-borne markers, which is similar to the results of our study. Positive effects of vitamin D supplement could be seen in patients aged 65 and older [28], in both upper and lower limb muscle function of healthy adults [23], and in lower limb muscle function of young athletes [29]. Therefore, there are two explanations for the finding of this study: The first one is that the participants in the current study were all professional athletes, who had undergone regular training for a period. We speculate that elite athletes are highly trained and have minimal margins for enhancement of muscle strength. The effect of long-term training may mask the effect of vitamin D deficiency on muscle. The second one is that we only measured the shoulder muscle profile of the participants, while vitamin D effect on other muscle groups, especially lower limb muscles, was ignored. Future study focused on lower limb muscle performance may be warranted to solve this issue.

We cannot suggest that vitamin D supplement increases rotator cuff strength per the findings of this study. However, it has already been established that a low vitamin D level increases the incidence of bone fractures and delays muscle recovery in athletes [8,10,21]. Therefore, while it is challenging to link vitamin D levels to athletic performance, it merits consideration.

Our study has a few limitations. First, external validity was limited. Since the participants of the study were limited to male volleyball athletes, the result of this study might not be applicable to other settings. Second, the vitamin D levels were not repeatedly confirmed on a long-term basis and were only examined one time. Third, factors that affect vitamin D levels, such as duration of exercise, degree of sun exposure, and diet, were not completely controlled. Though we only included young, healthy athlete and excluded those who regularly took vitamin D supplements and those with diseases interfering with the metabolism of vitamin D, bias should still be considered while applying the results of this study in practice.

## 5. Conclusions

According to the findings of the current study, the vitamin D level was insufficient in more than 70% of volleyball players. Though not being associated with isokinetic muscle weakness, vitamin D levels should be regularly monitored, and vitamin D should be given as a supplement to young elite athletes, considering its importance for musculoskeletal health.

## Figures and Tables

**Table 1:** Demographic characteristics of the study subjects.

Characteristics	Subjects (*n* = 52)
Age (year)	23.8 ± 2.8
Height (cm)	189.9 ± 8.3
Weight (kg)	82.7 ± 7.5
BMI (kg/m^2^)	22.8 ± 1.2
Vitamin D level (ng/mL)	25.2 ± 8.3
Position, no (%)	
Spiker	20 (38.7)
Blocker	11 (20.4)
Setter	11 (20.4)
Libero	10 (20.4)

Values are presented as means ± standard deviations. Abbreviation: BMI, body mass index.

**Table 2:** Correlation coefficients (*r*) between vitamin D level and other characteristics.

Characteristics	Vitamin D Level (ng/mL)	*p* Value
Age (year)	−0.315 *	0.02
Height (cm)	0.245	0.08
Weight (kg)	0.302	0.11
BMI (kg/m^2^)	−0.256	0.06
Shoulder muscle strength (ft/lb)		
60°/s		
External rotation	0.159	0.25
Internal rotation	0.245	0.08
180°/s		
External rotation	−0.093	0.51
Internal rotation	−0.037	0.79

* *p* < 0.05.

**Table 3:** Players demographics for each group of vitamin D status.

Variables	Vitamin D Status			*p* Value
	Deficiency (<20 ng/mL)	Insufficiency (20–30 ng/mL)	Sufficiency (>30 ng/mL)	
No. of players (%)	14 (26.9)	24 (46.2)	14 (26.9)	
Vitamin D level (ng/mL)	14.1 ± 3.4	25.5 ± 2.4	35.4 ± 4.0	<0.01 *
Age (year)	24.8 ± 3.5	23.6 ± 2.2	23.1 ± 2.9	0.05
Height (cm)	186.5 ± 8.9	191.6 ± 7.6	190.2 ± 8.3	0.17
Weight (kg)	81.3 ± 7.5	84.3 ± 7.2	81.2 ± 8.0	0.34
BMI (kg/m^2^)	23.3 ± 1.4	22.9 ± 1.1	22.4 ± 1.3	0.19

Values are presented as means ± standard deviations; * *p* < 0.05.

**Table 4:** Internal and external shoulder muscle strength according to vitamin D status.

Variable	Vitamin D Level			*p* Value
	Deficiency (<20 ng/mL)	Insufficiency (20–30 ng/mL)	Sufficiency (>30 ng/mL)	
No. players (%)	14 (26.9)	24 (46.2)	14 (26.9)	
60 degree/sec (ft/lb)				
External rotation	20.3 ± 3.1	21.4 ± 4.4	22.1 ± 4.0	0.51
Internal rotation	38.6 ± 6.6	41.4 ± 7.1	42.6 ± 8.8	0.34
180°/s (ft/lb)				
External rotation	18.7 ± 3.9	17.9 ± 4.1	17.5 ± 4.3	0.73
Internal rotation	36.0 ± 7.2	35.4 ± 7.9	35.2 ± 7.4	0.95

Values are presented as mean ± standard deviation.
